# 4513 European Ancestry as a Risk Factor for Atrial Fibrillation in Puerto Rican Hispanics

**DOI:** 10.1017/cts.2020.333

**Published:** 2020-07-29

**Authors:** Ariel Gonzalez-Cordero

**Affiliations:** 1 University of Puerto Rico-Medical Sciences Campus

## Abstract

OBJECTIVES/GOALS: Atrial fibrillation (AF) not only is the most common sustained cardiac arrhythmia in clinical practice placing patients at increased risk for thromboembolic events. Hispanics despite having a higher risk factor burden for developing AF have a lower overall incidence and prevalence of AF when compared to Non-HispanicWhites (NHW). European ancestry in the African American population was found to be an independent predictor for developing AF. Consequently, we have decided to evaluate if European ancestry is an independent risk factor for Puerto Rican Hispanics(PRH) METHODS/STUDY POPULATION: This project is a secondary analysis of a Puerto Rican population sample (n = 250) fromThePharmacogenetics of Warfarin in Puerto Ricans StudyandAGenomic Approach for Clopidogrel in CaribbeanHispanics; and1000GenomeProject to establish a control group of healthy PRH population. We will evaluate the presence of 111 known single nucleotide polymorphisms(SNP)associated with AF Europeans and determine the frequency in PRH population sample.Using admixture informatic markers (AIM) analysis will determine the percentage of admixture. Statistical analysis will include the use of Pearson Product-MomentCoefficient correlation analysis and multivariate regression. For admixture will use Maximum LikelihoodEstimation Markov Chain Monte Carlo models RESULTS/ANTICIPATED RESULTS: We anticipate that higher-frequency of AF associated European SNPs and overall higher percentage of European admixture will be associated with atrial fibrillation in PRH patients. DISCUSSION/SIGNIFICANCE OF IMPACT: The expected outcomes for this study are to identify the frequency of known genetic loci associated with AF Europeans and validate their use PRH population for machine learning risk factor models.

